# Diagnosis of Oral Hairy Leukoplakia: The Importance of EBV In Situ Hybridization

**DOI:** 10.1155/2017/3457479

**Published:** 2017-07-17

**Authors:** Luana L. Martins, José Henrique F. Rosseto, Natália Silva Andrade, Juliana Bertoldi Franco, Paulo Henrique Braz-Silva, Karem L. Ortega

**Affiliations:** ^1^Special Care Dentistry Center, Division of Oral and Maxillofacial Pathology, Department of Stomatology, School of Dentistry, University of Sao Paulo, Sao Paulo, SP, Brazil; ^2^Division of General Pathology, Department of Stomatology, School of Dentistry, University of Sao Paulo, Sao Paulo, SP, Brazil; ^3^Laboratory of Virology, Institute of Tropical Medicine of Sao Paulo, University of Sao Paulo, Sao Paulo, SP, Brazil

## Abstract

Oral hairy leukoplakia (OHL) is caused by the Epstein-Barr virus (EBV), which has been related to HIV infection. In situ hybridization (ISH) is the gold-standard diagnosis of OHL, but some authors believe in the possibility of performing the diagnosis based on clinical basis. The aim of this study is diagnose incipient lesions of OHL by EBV ISH of HIV-infected patients and the possible correlations with clinical characteristics of the patients. Ninety-four patients were examined and those presenting with clinical lesions compatible to OHL were submitted to biopsy prior to EBV ISH. Twenty-eight patients had lesions clinically compatible to the diagnosis of OHL, but only 20 lesions were confirmed by EBV ISH. The patients with OHL had a mean age of 41.9 years and were HIV-infected for 11.2 years, on average, including CD4 count of 504.7 cells/mm^3^ and log_10_ viral load = 1.1. Among the quantitative variables, there was a statistically significant correlation with age only (*P* = 0.030). In conclusion, the presence of OHL in patients with HIV/AIDS results in changes in the epidemiological characteristics of the disease, and this fact allied with subtle clinical-morphological features makes clinical diagnosis very difficult. Therefore, EBV ISH is important for a definitive diagnosis of OHL.

## 1. Introduction 

Approximately 60 percent of the HIV-infected individuals and 80 percent of those with acquired immunodeficiency syndrome (AIDS) present oral manifestations such as oral candidiasis (erythematous and pseudomembranous), oral hairy leukoplakia (OHL), Kaposi's sarcoma, non-Hodgkin's lymphoma, linear gingival erythema, necrotizing ulcerative gingivitis, and necrotizing ulcerative periodontitis [1, 2].

The presence of oral candidiasis and OHL within the oral cavity not only suggests HIV infection, but is possibly one of the first signs of development into AIDS in the HIV-infected individual [3, 4].

OHL represents an opportunistic infection related to the Epstein-Barr virus (EBV), being particularly present in patients infected by HIV [3, 5, 6]. Clinically, OHL is a white plaque with corrugated surface, painless, and not removable by scraping. According to Greenspan et al. “Puzzled by these findings, we tentatively applied to this lesion the name* oral hairy leukoplakia* (HL) because of the white color and corrugated or shaggy appearance of the lateral tongue seen many cases” [2]. This lesion is commonly found in the lateral border of the tongue. The histopathological characteristics are not exclusive to this lesion, which may include hyperkeratosis, epithelial hyperplasia, ballooning degeneration, and discrete or even absent inflammatory mononuclear cells infiltrate. For this reason, the criteria used for the final diagnosis of OHL have been always discussed [2, 7]. Previous study published by our group showed that among 36 cases of OHL diagnosed in clinical and histopathological basis, only 80.55% were EBV positive, confirming the previous diagnosis [8].

EBV can be detected by several techniques, such as polymerase chain reaction (PCR), immunohistochemistry, electron microscopy, and in situ hybridization (ISH), with the latter being considered the gold-standard exam [2, 8–13].

Therefore, the objective of this work is to diagnose incipience lesions of OHL by using in situ hybridization for identification of EBV within the oral mucosa of HIV-infected patients and to verify whether there is a relationship between the presence of OHL and clinical characteristics of the patients.

## 2. Materials and Methods

The present study was performed after approval by the ethics committee of the University of Sao Paulo Faculty of Dentistry, Sao Paulo, Brazil. The informed consent document was signed by all participants of the study, as dictated by the Declaration of Helsinki.

HIV-seropositive male and female patients older than 18 years attending the Special Care Dentistry Center (CAPE), School of Dentistry, University of Sao Paulo (FOUSP), were included in the present study, regardless of the use of antiretroviral therapy. CAPE is an outpatient dental clinic inside FOUSP facilities. All patients of CAPE were covered by the Brazilian public health system (SUS: Sistema Único de Saude). The patients having cognitive impairment (which does not allow the understanding of anamnesis and informed consent form), undergoing oncological treatments, or taking antiviral drugs (for herpes virus) in the past three months prior to evaluation were excluded from the study.

To calculate the sample size, we used the estimation of HIV-seropositive patients in CAPE-FOUSP in 2009 (1,867 patients). OHL prevalence considered was equal to 13.4 percent, which was previously reported in Brazilian study [14]. With a confidence interval of 90 percent, a power of sample of 80 percent, and a possible loss of 10 percent estimated for cross-sectional studies, the ideal sample size consisted of 97 patients. The sample size calculation was performed using the EpiInfo™ software (version 3.5.2; Centers for Disease Control and Prevention, Atlanta, GA, USA). The sample was consecutive; all patients that met the inclusion criteria and returned for routine visits from May 2010 to October 2010 were examined.

The patients were submitted to complete clinical examination, consisting of anamnesis and physical exam. Data on age, gender, smoking habits (cigarettes), ethnic group, HIV-exposure category, morbidities or chronic systemic diseases, duration of HIV-seropositivity, type and time of antiretroviral therapy, CD4 count, and viral load were collected. Physical exam was performed in a dental outpatient setting under artificial light and the patients were submitted to oral mucosa examination for identification and clinical diagnosis of oral manifestations related to HIV infection.

Biopsy was performed when there was clinical hypothesis of OHL. The biopsied material was fixed in 10% buffered formaldehyde solution before being sent to the Surgical Pathology Laboratory of the Oral Pathology Department of the University of Sao Paulo Faculty of Dentistry. Histological sections were blocked into paraffin and stained with Hematoxylin and Eosin (H&E) according to standard laboratory procedure, and in situ hybridization was performed for identification of EBV according our previous published work [8].

The histological diagnoses of all lesions were performed by an expert oral pathologist and the characteristics considered to diagnose OHL were the same as those described by Greenspan et al. (2016), Khammissa et al. (2016), and Braz-Silva et al. (2008) [2, 6, 8].

The data were analyzed using the EpiInfo software (Centers for Disease Control and Prevention, Atlanta, GA, USA). Descriptive statistics were used for univariate analysis. In bivariate analysis, qualitative variables [e.g., gender, CDC classification, and smoking habits (cigarettes)] were assessed by using Fisher's exact test. To compare the quantitative variables (age, time of infection, lowest CD4 count, current CD4 count, HIV-viral load, duration of ARVT, and current duration of ARVT) and the presence of OHL, we applied the nonparametric Mann–Whitney *U* test, because these variables showed a nonnormal distribution by the Kolmogorov-Smirnov test. In all analyses, the significance level was fixed as alpha = 5%. This analysis was used to reveal potential associations between clinical characteristics of the patient and the presence of OHL.

## 3. Results

A total of 94 HIV-seropositive patients were included in the present study representing 96.9% of the ideal sample, in which 68 (72.3%) were males and 26 (27.7%) females, with mean age of 45.2 years. With regard to skin color, 53 (56.4%) were classified as white, 23 (24.5%) as black, and 18 (19.1%) as mulatto. Evaluation of deleterious oral habits revealed that 29.8% of the patients were current smokers, 24.5% were former smokers, and 20.2% drank alcoholic beverages regularly. The patients were also classified according to the HIV-exposure category reported: three patients (3.2%) contracted HIV by using injectable drugs, 42 (44.7%) by heterosexual relation, 39 (41.5%) by homosexual relation, and two (2.1%) by vertical transmission and eight (8.5%) did not know about the probable route of contamination. The mean duration of HIV infection was 12.4 years, with 63.8% developing AIDS (36.2% were in category B2 and 63.8% were in category C3 of the 1993 Revised Classification System for HIV Infection and Expanded Surveillance Case Definition for AIDS Among Adolescents and Adults). All patients were on antiretroviral therapy.

Medical history revealed that 68 patients had been suffering from morbidities [systemic arterial hypertension (18), diabetes mellitus type 2 (8), anemia (23), viral hepatitis (24), and hypercholesterolemia (4)] and 73 from systemic opportunistic infections [toxoplasmosis (9), leishmaniasis (1), pneumonia (36), tuberculosis (27), meningitis (7), syphilis (23), and gonorrhea (10)] during the clinical course of the HIV disease. Eighty-eight patients (89.7%) were using antiretroviral therapy for 9.4 years, on average, whereas the current therapeutic regimen lasted 3.8 years, on average.

On the intraoral clinical examination, patients were diagnosed with oral manifestations of HIV infection such as candidiasis (*n* = 5), herpes labialis (*n* = 1), aphthous ulcers (*n* = 1), necrotizing gingivitis (*n* = 1), and angular cheilitis (*n* = 1).

**Figure 1:**
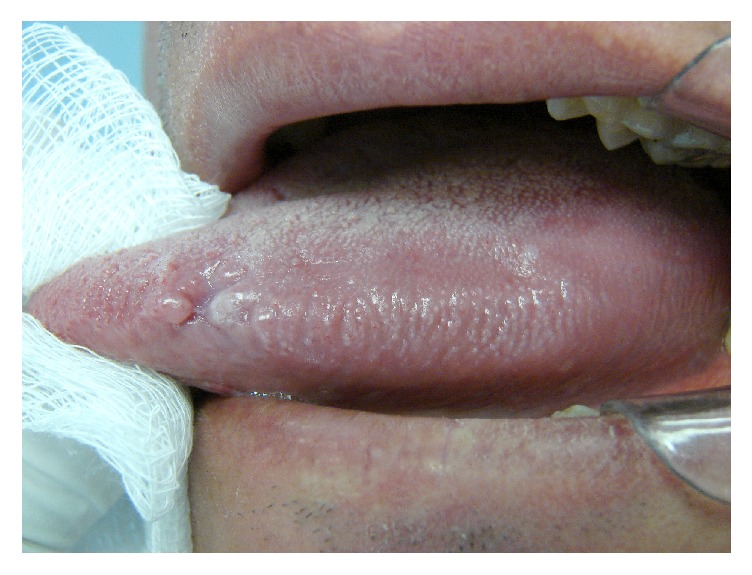
Clinical aspect of the oral hairy leukoplakia.

Twenty-eight patients were clinically suspected of having OHL and underwent biopsy for confirmation of diagnosis. Clinically, none of the lesions showed expressive increase in the epithelial keratinization or significant elevation of the affected mucosa (Figure 1). The histological characteristics taken into consideration were the following: acanthosis, epithelial hyperplasia, cells resembling koilocytes, cells with ground glass nuclei and perinuclear halo, and absent or discrete inflammatory infiltrate in the surrounding connective tissue. The lamina propria consisted of dense connective tissue, in some cases with both presence and absence of a few inflammatory mononuclear cells.

**Figure 2:**
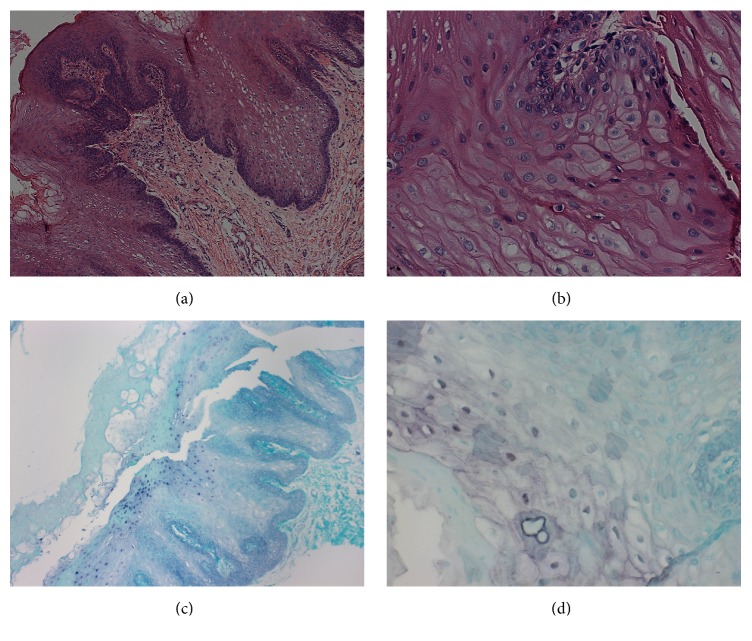
Histological characteristics of OHL and in situ hybridization reaction. (a) Histopathological section, stained with H&E, showing stratified squamous epithelium, acanthosis, epithelial hyperplasia, cells resembling koilocytes, cells with opaque nucleus, and perinuclear halo (100x magnification). (b) Section stained with H&E showing cells resembling koilocytes (400x magnification). (c) In situ hybridization reaction with positivity for EBV (100x magnification). (d) In situ hybridization reaction with positivity for EBV (400x magnification).

Both histological examination and in situ hybridization confirmed the clinical diagnosis in 20 patients (71.42% of the clinically diagnosed patients), with the lateral border of the tongue being the only site where these lesions were found (Figure 2). Cohen's kappa coefficient between clinical examination and in situ hybridization was 0.78.

The white lesions in eight EBV negative lesions were diagnosed as irritative hyperkeratosis.

The qualitative variables (i.e., gender, CDC classification, and smoking) were assessed by using the Fisher's exact test and no statistically significant correlation was found between the variables and presence of OHL. With regard to quantitative variables, statistically significant association was found only between patient age and presence of OHL (*P* = 0.030), with the mean age of patients with OHL being lower than that of those without it (Table 1). The age of patients with OHL ranged from 19 to 51 and the age of HIV patients without OHL lesions ranged from 18 to 69 years.

## 4. Discussion

By analyzing the 2015 HIV/AIDS Epidemiological Bulletin, it was found that in Brazil there are 830,000 people living with HIV and 44,000 new cases were registered only in 2015. The highest prevalence is observed in male individuals, with an increase among homosexual men. Another important observation is the increase in cases of AIDS in the age groups of 15–19 and 20–24 years [15, 16].

In our study group, the high incidence of opportunistic systemic infections linked to the immunosuppression caused by HIV reflects the clinical staging of the disease, thus contributing to the diagnosis for these patients [17]. On the other hand, the systemic morbidities presented by HIV+ patients on this study may be reflecting the adverse effect of the long-term use of medication, since the patients had been taking it for more than nine years, on average. Although the highly active antiretroviral therapy (HAART) has extraordinarily contributed to increasing the life expectancy and to improving the quality of life of HIV/AIDS patients, today it is known that this treatment has also accounted for the emergence of several adverse reactions in these patients like hematological, gastrointestinal, hepatic, and metabolic effects, among others. In addition, these patients present symptoms involving nausea, diarrhea, hyperlipidemia, dyslipidemia, neuropathy, allergic reactions, myocardial infarction, diabetes, myalgia, lactic acidosis, nephropathies, osteoporosis, and osteopenia [18].

We believe that the current therapeutic regimen of the studied patients was demonstrated to be effective as the patients showed suitable virological and immunological responses (CD4 count > 500 and CV log < 1). According to the literature, low viral loads and high CD4 counts reflect clinically the decreased amount of opportunistic oral lesions [19] found in the patients. Our results confirm the literature data. In a previous study published by our group, the prevalence of oral manifestations was 53.29% in patients with mean CD4 count of 160 cells/mm^3^. In the present study, the prevalence of patients with oral lesions dropped to 22.34% [14, 20].

Lesions of OHL were identified in 21.27% of the patients evaluated. This figure does not reflect the decrease reported by other studies on prevalence after use of HAART [21, 22], all indicating a marked drop in the prevalence of OHL. Despite the reduced incidence, OHL was found to be the most refractory lesion in the antiretroviral therapy as although the number of occurrences decreased over the years, it was not gradual and constant as in the case of candidiasis and SK [14]. In our study, we have observed that the clinical characteristics of OHL were different compared to the historical pattern found in our outpatient unit: from an ample, flower-shaped lesion occupying a large area of the lateral border of the tongue to a much more discrete lesion with clinical characteristics less exacerbated than those found at the beginning of the epidemics. However, because many lesions have not been diagnosed, it is necessary to perform both biopsy and in situ hybridization for a correct diagnosis. Gold-standard laboratory exams should also be performed for the diagnosis of OHL, which became clear in our work. Some recently published studies have showed the usefulness of the cytopathology to diagnosis of the OHL, always associated with molecular tools to EBV detection, opening a new perspective to use less invasive diagnostic methods for this condition [23, 24].

Some researchers also state that the presence of this lesion could indicate a therapeutic failure or serve as a marker of AIDS progression [4, 7, 9, 25]. In our case, it was not possible to correlate the presence of OHL with an occasional failure of the treatment because patients with OHL had a viral load of 1.1 log (*P* = 0.155), on average, or consider the lesion as a marker of AIDS progression because the correlation between presence of OHL and disease staging was not statistically significant (*P* = 0.301). The high CD4 count in patients with OHL (mean value of 504.7 cells/mm^3^) was very close to the mean value of patients without OHL (504.7 cells/mm^3^) (*P* = 0.726) and also differed from the literature, which always correlated the lesion with low TCD4 counts [14, 20, 25]. It was not possible to correlate the presence of OHL with either the use of tobacco (*P* = 1.000), time of HIV infection (*P* = 0.239), or duration of antiretroviral therapy (*P* = 0.36).

## 5. Conclusion

The clinical aspects of OHL in patients with HIV/AIDS have been changed in the past years, making the diagnosis based only on clinical basis very difficult. The use of in situ hybridization for EBV detection has been fundamental for achieving a definitive diagnosis of OHL, mainly in patients with oral lesions out of the classic patterns.

## Figures and Tables

**Table 1:** Comparison between presence of oral hairy leukoplakia and quantitative variables of the patients.

Variable	Oral hairy leukoplakia	*n*	Mean	SD	Median	*U*	*P* ^*∗*^
Age (years)	Yes	20	41.9	6.8	42.5	505	**0.030**
	No	74	46.1	10.7	47.0		
Time of infection (years)	Yes	20	11.2	5.5	11.0	613	0.239
	No	74	12.8	4.9	13.0		
Lowest CD4 count (cells/mm^3^)	Yes	20	197.4	200.8	150.0	648.5	0.446
	No	73	212.4	168.4	200.0		
Current CD4 count (cells/mm^3^)	Yes	20	504.7	243.1	507.0	692.5	0.726
	No	73	544.5	301.4	500.0		
HIV-viral load (log copies/ml)	Yes	20	1.1	1.9	0.0	625	0.155
	No	73	0.6	1.4	0.0		
Duration of ARVT (years)	Yes	20	8.8	5.0	8.5	606	0.360
	No	70	9.6	4.6	10.0		
Current duration of ARVT (years)	Yes	20	2.9	2.7	2.0	554	0.154
	No	70	4.1	3.6	3.5		

^*∗*^Mann–Whitney *U* test. SD: standard deviation; ARVT: antiretroviral therapy.
